# G Protein-Coupled Estrogen Receptor Regulates Actin Cytoskeleton Dynamics to Impair Cell Polarization

**DOI:** 10.3389/fcell.2020.592628

**Published:** 2020-10-22

**Authors:** Dariusz Lachowski, Ernesto Cortes, Carlos Matellan, Alistair Rice, David A. Lee, Stephen D. Thorpe, Armando E. del Río Hernández

**Affiliations:** ^1^Cellular and Molecular Biomechanics Laboratory, Department of Bioengineering, Imperial College London, London, United Kingdom; ^2^Institute of Bioengineering, School of Engineering and Material Science, Queen Mary University of London, London, United Kingdom; ^3^UCD School of Medicine, UCD Conway Institute of Biomolecular and Biomedical Research, University College Dublin, Dublin, Ireland

**Keywords:** actin cytoskeleton, focal adhesions, cell polarization, mechanosensing, RhoA, G protein-coupled receptors

## Abstract

Mechanical forces regulate cell functions through multiple pathways. G protein-coupled estrogen receptor (GPER) is a seven-transmembrane receptor that is ubiquitously expressed across tissues and mediates the acute cellular response to estrogens. Here, we demonstrate an unidentified role of GPER as a cellular mechanoregulator. G protein-coupled estrogen receptor signaling controls the assembly of stress fibers, the dynamics of the associated focal adhesions, and cell polarization *via* RhoA GTPase (RhoA). G protein-coupled estrogen receptor activation inhibits F-actin polymerization and subsequently triggers a negative feedback that transcriptionally suppresses the expression of monomeric G-actin. Given the broad expression of GPER and the range of cytoskeletal changes modulated by this receptor, our findings position GPER as a key player in mechanotransduction.

## Introduction

The G protein-coupled estrogen receptor (GPER) belongs to the heptahelical transmembrane family of G protein-coupled receptors (GPCRs) and initiates rapid signaling cascades in response to both endogenous estrogens such as 17β-estradiol and man-made compounds (Revankar et al., 2005; Prossnitz and Barton, 2011). These GPER-mediated events may involve the generation of second messengers such as Ca^2+^, as well as the activation of protein kinase A and tyrosine kinase receptors, among others. Given that GPER is broadly expressed in eukaryotic cells and because of its potential to regulate multiple downstream signaling, including cell survival and proliferation, GPER has attracted significant attention in biology and medicine in the last 20 years (Zimmerman et al., 2016; Barton et al., 2018; Hilger et al., 2018).

The small Rho GTPases are molecular switches downstream of GPCR that control a plethora of biological signaling in eukaryotic cells. They achieve this control by cycling between the GTP-active and GDP-inactive states (Etienne-Manneville and Hall, 2002). The RhoA GTPase (RhoA) is one of the most prominent members of the Rho GTPase family, which controls and shapes actin cytoskeleton by promoting actin polymerization *via* formins (mDia), and through actomyosin contractility by triggering the phosphorylation of the regulatory myosin light chain-2 (MLC-2) *via* Rho kinase (ROCK; Sadok and Marshall, 2014). This RhoA-dependent induction of cytoskeletal contractility is required for the nuclear translocation and activation of the transcriptional factor yes-associated protein 1 (YAP), a mechanotransducer that has cardinal roles in development, tissue homeostasis (Dupont et al., 2011), cancer (Calvo et al., 2013), and cardiovascular diseases (Wang et al., 2016). Yes-associated protein 1 activation influences further mechanical processes including genomic regulation of focal adhesion formation (Nardone et al., 2017).

The actin cytoskeleton is a complex and highly dynamic network of protein filaments that determines cell morphology, maintains the mechanical integrity of the cell, transmits forces, remodels in response to stimuli, and polarizes to enable cell migration (Krishnan et al., 2009; Pollard and Cooper, 2009; Gardel et al., 2010; Maruthamuthu et al., 2010). Actin monomers (G-actin) polymerize into actin filaments (F-actin), which in turn organize into bundles known as stress fibers (Pellegrin and Mellor, 2007). The assembly of actin filaments is controlled by two key cytoskeletal regulators, mDia and the Arp2/3 complex. The formin mDia, which is a downstream effector of RhoA, guides the formation of linear actin filaments by nucleating the polymerization of actin filaments *de novo*. Conversely, the Arp2/3 complex binds to preexisting actin filaments and nucleates the polymerization of daughter filaments at a constant 70° angle, resulting in a branched actin network (Mullins et al., 1998). The structure and assembly kinetics of actin stress fibers dominate many dynamic cellular processes such as (i) spreading, adhesion, contraction, locomotion, and mechanosensing (Tojkander et al., 2012; Murrell et al., 2015); (ii) the fate of stem cells (McBeath et al., 2004); and (iii) collective cell migration in morphogenesis and cancer (Friedl and Gilmour, 2009; Ilina and Friedl, 2009). The association of these actin stress fibers with myosin (actomyosin) constitutes the primary contractile machinery of the cell (Tojkander et al., 2012). This cytoskeletal machinery, linked to a dynamic population of focal adhesions, enables cells to sense and interact mechanically with their microenvironment (Ohashi et al., 2017).

Here, we demonstrate that RhoA-mediated GPER signaling can regulate the structure and dynamics of the actin cytoskeleton in fibroblasts. We observe that GPER activation decreases the number and thickness of stress fibers, the stiffness of the cytoskeleton, and the size and number of focal adhesions. Then, we use fluorescence recovery after photobleaching (FRAP) to quantify focal adhesion turnover and actin polymerization rates and demonstrate that GPER signaling impairs actin filament assembly as well as actin branching and cell polarization. Finally, we demonstrate that GPER downregulates actin expression in a RhoA-dependent manner.

## Materials and Methods

### Cell Culture, Transfection, and Antibodies

Human foreskin fibroblasts (HFFs) were from the ATCC (catalog number SCRC-1041). Mouse embryonic fibroblasts (MEFs) were a gift from Dr. Wolfgang Ziegler and have been previously described by Xu et al. (1998). Both cell lines were maintained in high-glucose DMEM supplemented with 10% v/v FBS and 1% v/v GlutaMax (Thermo Fisher Scientific, United States), 1% v/v penicillin/streptomycin (Sigma Aldrich, P4333), and 1% v/v Fungizone/amphotericin B (Gibco, 15290-026). A humidified 37°C incubator with 5% CO_2_ was used for culturing both cell lines. Cells were negative when tested for mycoplasma contamination. The primary antibodies used in the experiments were total RhoA (Millipore, 04-822, 1/1,000 dilution), pRhoA (Abcam, ab41435, 1/1,000), paxillin (BD Biosciences, 612405, 1/200), β-actin (Abcam, ab8226, 1/10,000), GPER (Abcam, ab39742, 1/100), and anti-Arp3 antibody (Abcam, ab49671, 1/100). The secondary antibodies used in the experiments were anti-mouse HRP (Invitrogen, 626580, 1/2,000), anti-rabbit HRP (Abcam, ab137914, 1/2,000), anti-mouse Alexa-488 (Invitrogen, A11029, 1/400), anti-rabbit IgG (H+L) Alexa-488 (Invitrogen, A11034, 1/400), IRDye 680RD donkey anti-mouse IgG (H+L) (LI-COR 925-68072, 1/15,000), or IRDye 800CW donkey anti-rabbit IgG (H+L) (LI-COR 925-32213, 1/15,000). siRNA targeting GPER was purchased from Santa Cruz Biotechnology (sc-60743). G protein-coupled estrogen receptor agonist (G1) and GPER antagonist (G15) were purchased from Tocris and used at 1 μM: G1 (Tocris, 2577) and G15 (Tocris, 3678) in treatments of 24 h unless specifically indicated. CellLight^TM^ Actin-GFP, BacMam 2.0 (Thermo Fisher Scientific, C10506) was used for fluorescence recovery after the photobleaching experiments. pRK GFP paxillin plasmid, also used for FRAP, was a gift from Kenneth Yamada (Addgene plasmid #50529). The constitutively active RhoA plasmid (pRK5-myc-RhoA-Q63L) was a gift from Gary Bokoch (Addgene plasmid #12964). This plasmid was used as a template to create the plasmid RhoA (S188A/Q63L) by substitution of the serine amino acid in position 188 to alanine using site-directed mutagenesis. Constitutively active mDia1 (mDia1ΔN3—an FH1-FH2 unit mutant) plasmid was a gift from Alexander Bershadsky, and GFP-cortactin was a gift from Anna Huttenlocher (Addgene plasmid #26722).

### Scanning Electron Microscopy

The morphology of the cells was analyzed using scanning electron microscopy (SEM). Cells were fixed with 3% v/v EM-grade glutaraldehyde in 0.1 M PBS for 15 min at 37°C and washed with 0.1 M PBS. Following fixation, cells were lipid contrast stained using 1% w/v OsO_4_ in PBS for 1 h at room temperature and dehydrated in ethanol with gradually increasing concentration. Samples were air dried overnight and coated with 10 nm of chromium. The images were acquired using Zeiss Auriga Cross Beam SEM with 7.5 × 10^3^ magnification, 5 kV. Images were analyzed using FIJI by thresholding in order to detect the outline of at least 10 cells per condition. The obtained masks were quantified using the area and roundness parameters.

### Immunofluorescence Staining

Cell immunofluorescence staining was done on coverslips coated with 10 μg/ml fibronectin in PBS (Gibco, PHE0023). Following pertinent treatment, cells were fixed with 4% w/v paraformaldehyde (Sigma, P6148) in D-PBS (Sigma, D8537) for 10 min, permeabilized with 0.5% w/v saponin (Sigma, 47036), and then blocked with 1% w/v BSA (Sigma, A8022) and 22.52 mg/ml glycine (Sigma, G8898) in PBST for 30 min. After blocking, cells were incubated with primary antibodies prepared in blocking solution overnight at 4°C in a humidified chamber. Then, cells were washed in D-PBS and incubated with Alexa Fluor 488-conjugated secondary antibodies and phalloidin (Invitrogen, A22283, 1/500) prepared in PBS for 1 h at room temperature. Finally, coverslips were washed in PBS and mounted in mounting reagent with 4,6-diamidino-2-phenylindole (Invitrogen, P36931). Widefield fluorescent images were taken with Nikon Ti-e Inverted Microscope (Ti Eclipse, C-LHGFI HG Lamp, CFI Plan Fluor 40 × NA 0.6 air objective; Nikon; Neo sCMOS camera; Andor) with NIS elements AR software. Staining intensity was measured in Fiji (Schindelin et al., 2012) using the “mean gray value” parameter applied to a region of interest (ROI) created for manually segmented cells based on DIC images. Mean gray values for each image’s background were subtracted for each measured staining intensity.

Ventral stress fibers were identified by overlaying widefield images of actin and paxillin, then selecting actin fibers attached to focal adhesions at both ends. Number per μm^2^ was calculated by dividing manually counted number of ventral stress fibers by the cell area measured from brightfield images. The thickness of these fibers was quantified in Fiji by using the *plot profile* function for a straight line overlaid perpendicular in the middle of each ventral stress fiber and measuring the peak width of the mean gray value of actin widefield image. Arp3 edge to center staining intensity was quantified as a ratio of mean gray value (intensity) of the signal within the outer 5 μm of a whole-cell ROI and mean gray value of the signal within the inner ROI (outer 5 μm ROI subtracted from the whole-cell ROI).

### Atomic Force Microscopy

Measurements of cell compliance were conducted on a Nanowizard-1 (JPK Instruments, Berlin, Germany) atomic force microscope operating in force spectroscopy mode mounted on an inverted optical microscope (IX-81; Olympus, Tokyo, Japan). Atomic force microscopy (AFM) pyramidal cantilevers (MLCT; Bruker, Camarillo, CA, United States) with a spring constant of 0.03 N/m (nominal stiffness reported by the manufacturer) were used with a 15-μm diameter polystyrene bead attached at room temperature. Before conducting measurements, cantilever sensitivity was calculated by measuring the force–distance slope in the AFM software on an empty petri dish region. Cells were seeded on fibronectin-coated glass fluorodishes and allowed to spread for >2 h. Cell attachment to the substrate was confirmed by visual inspection before conducting the nanoindentation procedure. For each cell analyzed, force curves were acquired at an approach speed of 5 μm/s and a maximum set point of 1 nN. Force curves were taken in regions distal from the cell nucleus to avoid assessing nuclear stiffness. The force–distance curves were used to calculate elastic moduli in the AFM software through the application of the Hertz contact model (Harris and Charras, 2011).

### Fluorescence Recovery After Photobleaching

The FRAP experiments were conducted on glass-bottomed petri dishes (Mattek) coated with human plasma FN (10 μg/ml in PBS; Gibco, PHE0023) and incubated at 37°C. Six hours after seeding, cells were transfected either with pRK-GFP-paxillin by electroporation using the Neon Transfection system (Thermo Fisher Scientific) with one pulse of 1,300 V for 30 ms or with CellLight^TM^ actin-GFP; 2 μl of the reagent was added to 2 ml of the complete cell culture medium per dish and added to the cells. Confocal photobleaching was carried out 24 h after the transfection using an inverted microscope (Ti Eclipse, C2-SHS C2si Ready Scanner, Ti-TIRF-E Motorized TIRF Illuminator, CFI Plan Apo TIRF 60 × NA 1.49 oil objective; Nikon). Five confocal images were taken at 5 s intervals prior to bleaching for reference. Specified regions of the cells were then bleached using the confocal laser at 100% power. Images were taken at 5 s intervals for 100 s to capture fluorescent recovery. Images were analyzed with FIJI (measured mean gray value for each bleached ROI for each time point), with the fluorescent signal normalized between the prebleach intensity and background. Statistical analysis was then carried out using Prism (GraphPad). Data was pooled from repeats. Fluorescence recovery curves were compared using extra sum-of-squares *F* test on the best fit lines. Immobile fraction was calculated as 1 *-* plateau for each curve. Error bars represent the standard error for each plateau. Half time of recovery (*t*_1/2_) was calculated separately for curves fit for each dataset and represented as mean for each condition with standard error bars.

### RT-PCR

Total RNA was extracted using the RNeasy Mini kit (Qiagen, 74104), and 1 μg of total RNA was reverse-transcribed using the High-Capacity RNA-to-cDNA kit (Applied Biosystems, 4387406) according to the manufacturer’s instructions. qPCR was performed using the SYBR Green PCR Master Mix (Applied Biosystems, 4309155) with 100 ng cDNA input in 20 μl of reaction volume. RPL0 (60S acidic ribosomal protein) expression level was used for normalization as a housekeeping gene. The primer sequences were as follows: RPLP0 (F) 5′-CGGTTTCTGATTGGCTAC-3′, RPLP0 (R) 5′-ACGATGTCACTTCCACG-3′; MLC-2: forward, 5′-ATCCACC TCCATCTTCTT-3′ and reverse, 5′-AATACACGACCTCC TGTT-3′; CTGF: forward, 5′-TTAAGAAGGGCAAAAAGTGC-3′ and reverse, 5′-CATACTCCACAGAATTTAGCTC-3′; ANKDR1: forward, 5′-TGAGTATAAACGGACAGCTC-3′ and reverse, 5′-TATCACGGAATTCGATCTGG-3′; and ACTB (β-actin): forward, 5′-GACGACATGGAGAAAATCTG-3′ and reverse, 5′-ATGATCTGGGTCATCTTCTC-3′. All primers were used at 300 nM final concentration. The relative gene expression was analyzed by comparative 2^–ΔΔ*Ct*^ method.

### Western Blotting

Cells were washed with chilled PBS and lysed in radio immunoprecipitation assay (RIPA) buffer containing Halt protease and phosphatase inhibitors (Thermo Fisher Scientific). Lysate was collected using a cell scraper, disrupted by repetitive trituration through a 25-gauge needle, and incubated for 30 min on ice with periodic mixing. This was followed by centrifugation at 12,000 *g* for 20 min at 4°C. The protein concentration in the supernatant was determined using a BCA protein assay kit (Fisher Scientific, 23225). Cell lysates were mixed with 4× Laemmli buffer (Bio-Rad, 1610747) including β-mercaptoethanol and denatured by heating at 95°C for 5 min. Samples were loaded into a 4–20% Mini-PROTEAN TGX Precast Gel (Bio-Rad, 4561096), and proteins were transferred to nitrocellulose membranes (Bio-Rad). Protein was stained using REVERT total protein stain (LI-COR, 926-11010) as per the manufacturer’s instructions, and blots were imaged using an Odyssey infrared imaging system (LI-COR). The stain was removed using REVERT Reversal Solution (LI-COR, 926-11013), followed by washing in tris-buffered saline (TBS). The membranes were blocked in Odyssey blocking buffer (LI-COR, 927-50000) for 1 h followed by overnight incubation with primary antibodies in 0.1% v/v Tween-20 in TBS (TBST). After further washes in TBST, blots were incubated for 1 h with secondary antibodies. Membranes were washed again in TBST and imaged using an Odyssey infrared imaging system (LI-COR). Total protein for normalization and target protein expression were quantified using Image Studio Lite (Version 5.2, LI-COR). Target protein was normalized to total protein per lane and presented relative to the control group.

### G-LISA Assay for RhoA

The intracellular amounts of total RhoA and RhoA-GTP were determined by using the total RhoA ELISA and G protein-linked (G-LISA) assays (Cytoskeleton, BK124) according to the manufacturer’s instructions. Briefly, cells were washed with cold PBS and homogenized gently in ice-cold lysis buffer. Twenty microliters was removed for protein quantification in order to adjust sample concentration to 0.5 mg/ml. After adding an equal volume of binding buffer, triplicate assays were performed using 1.5 μg of protein per well. Samples were incubated for 30 min and then washed three times with washing buffer. Antigen-presenting buffer was added for 2 min before removal; samples were then incubated with 1/250 dilution of anti-RhoA antibody at room temperature for 45 min, washed three times, and incubated with secondary antibodies for another 45 min. HRP detection reagent was added, and signal was read by measuring absorbance at 490 nm using a microplate spectrometer.

### Statistical Analysis

All statistical analyses were conducted with the Prism software (version 8, GraphPad). Data were generated from multiple repeats of different biological experiments to obtain the mean values and SEM displayed throughout. *P* values have been obtained through *t* tests on unpaired samples with parametric tests used for data with a normal distribution. ANOVA and *post hoc* Dunnett’s test were used to perform multiple comparison test on normally distributed data, and Kruskal–Wallis test was used for multiple comparison of non-normally distributed data. Significance was set at *P* < 0.05 where graphs show significance through symbols (^∗^0.01 < *P* < 0.05; ^∗∗^0.001 < *P* < 0.01; ^∗∗∗^0.0001 < *P* < 0.001; ^****^P < 0.0001).

## Results

### GPER Inhibits RhoA Activation in Fibroblasts

**FIGURE 1:**
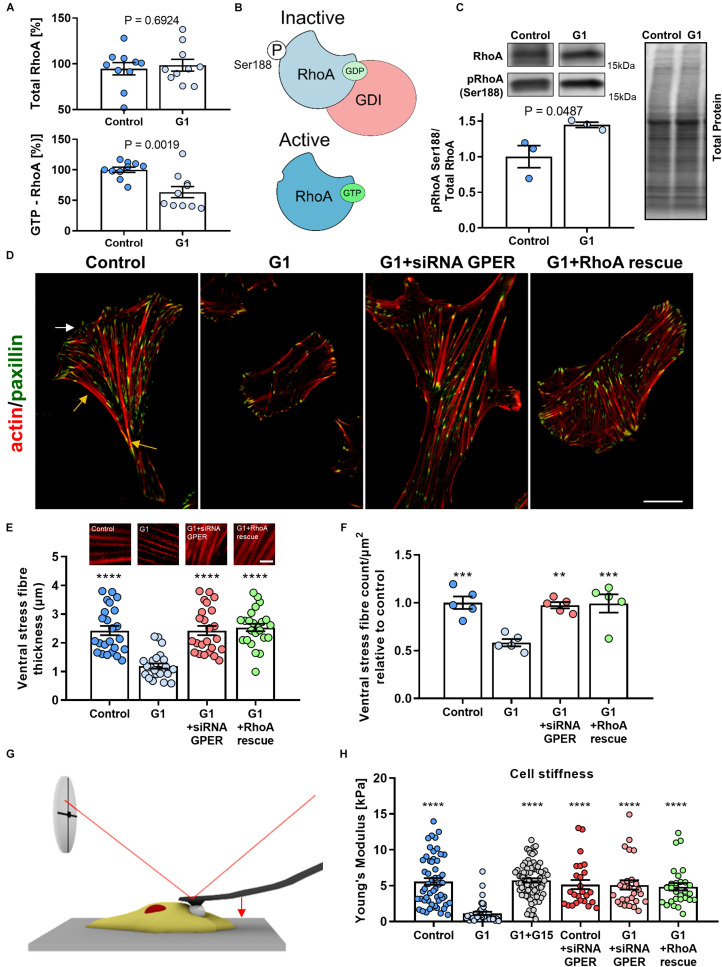
Actin fiber thickness and cell compliance are dependent on the G protein-coupled estrogen receptor (GPER)/RhoA GTPase (RhoA) axis. **(A)** Quantification of total and active GTP-bound RhoA, normalized to the control condition measured by G protein-linked (G-LISA) assay in human foreskin fibroblasts (HFFs) treated with GPER agonist G1 or vehicle control. **(B)** Schematics of the mechanism of RhoA regulation. Phosphorylation on the serine 188 residue increases the affinity between GDP-RhoA and guanine nucleotide dissociation factor (GDI), sequestering inactive RhoA from the cytoplasm and preventing its activation. **(C)** Western blot quantification of pRhoA Ser188 (inactive RhoA) normalized to total RhoA. Three biological samples run in triplicate. *t* test *P* values provided. **(D)** Representative images of HFFs in control, G1, G1+siRNA GPER knockdown, or G1+RhoA rescue using constitutively active RhoA (S188A/Q63L). The white arrow indicates the lamellipodium and the yellow arrows the localization of the ventral stress fibers. Scale bar is 20 μm. **(E,F)** Quantification of ventral stress fiber thickness and count per μm^2^ in HFFs with representative images of actin fibers. Scale bar represents 5 μm. **(G)** Schematic image of cell cytoskeletal stiffness measurements with atomic force microscopy (AFM). **(H)** Mean cell Young’s modulus as determined by AFM for control, G1, G1 + G15, control + siRNA GPER, G1 + siRNA GPER, and G1+RhoA rescue: *n* = 55, 41, 78, 25, 30, and 28 cells, respectively. Histogram bars represent mean ± SEM; dots represent individual data points. Three experimental replicates. Markers denote significant difference from G1 condition by ANOVA with Dunnett’s *post hoc* test, **0.001 < *P* < 0.01, ***0.0001 < *P* < 0.001, ****P < 0.0001.

Previous work has demonstrated that GPER signaling can inhibit RhoA activation (Yu et al., 2017; Cortes et al., 2019c, d). We used immunoassays to measure activated (GTP-bound) and total levels of RhoA and observed a significant 40% decrease in the levels of GTP-bound (active) RhoA in HFFs treated with the selective GPER agonist (Bologa et al., 2006) G1 compared with control HFFs, whereas no significant change in total RhoA was observed between the control and G1-treated HFFs (Figure 1A). These results indicate that GPER activation does not affect the expression of RhoA but instead inhibits its activation. RhoA activation is regulated by a variety of factors: guanine nucleotide exchange factors (GEFs) activate RhoA by promoting the exchange of GDP by GTP, while GTPase-activating proteins (GAPs) catalyze the substitution of GTP by GDP leading to the inactivation of RhoA. Furthermore, the inactive pool of GDP-bound RhoA is sequestered in the cytosol through the formation of a complex with guanine nucleotide dissociation factors (GDIs), which prevents RhoA activation (Ellerbroek et al., 2003; Lessey et al., 2012; Figure 1B). Using Western blot, we confirmed that there was no change in the total levels of RhoA between the control and G1-treated HFFs, and observed around 45% increase in the levels of RhoA phosphorylated in serine 188 (pRhoA-Ser188, inactive) in G1-treated HFFs compared with control HFFs (Figure 1C and Supplementary Figure 1). It is well documented that phosphorylation of the serine residue 188 in the C-terminal tail of RhoA increases the affinity of the RhoA-GDI complex, preventing its dissociation and thereby promoting RhoA inactivation (Lang et al., 1996; Forget et al., 2002; Ellerbroek et al., 2003). Our results point toward this mechanism of RhoA inhibition mediated by RhoGDI, which is in turn consistent with the mechanism of GPER-mediated RhoA inhibition observed previously (Yu et al., 2014, 2017).

### GPER Regulates Actin Cytoskeleton Organization

Given the central role of the actin cytoskeleton in cellular mechanical activity, we sought to investigate the effect of GPER activation on the assembly and organization of actin stress fibers. First, we confirmed that GPER is expressed in HFFs and MEFs (Supplementary Figure 2A). Then, we characterize the thickness and number (normalized by cell area to account for changes in cell morphology) of ventral stress fibers using immunofluorescence microscopy. Ventral stress fibers are a subset of actin stress fibers that attach to focal adhesions at both ends and contain myosin II, making them the primary contractile machinery of many cells. The abundance of ventral stress fibers is therefore a hallmark of highly contractile and mechanically active fibroblasts (Tojkander et al., 2012). In control HFFs, we observed numerous and thick ventral fibers, with an average of 2.4 ± 0.2 μm thickness (mean ± SEM, *n* = 24) (Figures 1D,E). These fibers were widely distributed across the entire cell body and particularly abundant in the posterior area of the well-polarized cells and less numerous at the leading edge (common localization of ventral fibers) (Tojkander et al., 2012). In contrast, G1-treated HFFs showed a more uniform distribution of stress fibers and a significant decrease in the thickness and number (normalized by cell area) of ventral fibers, with an average thickness of 1.2 ± 0.1 μm (mean ± SEM, *n* = 24) and an ∼40% decrease in the number of stress fibers compared with control HFFs (Figures 1E,F). In addition, we observed that G1 treatment did not affect the thickness and number of ventral fibers in HFFs that were previously treated with siRNA to knock down GPER expression or expressing the constitutively active form of RhoA (S188A/Q63L) (Figures 1E,F and Supplementary Figure 2B), indicating that the mechanism of stress fiber regulation is dependent on the GPER–RhoA axis. Similarly, analysis of cell morphology revealed profound changes in cell area and shape in response to GPER activation. Using SEM, we observed that G1-treated HFFs and MEFs had a significantly smaller contact area and were significantly rounder than control cells (Supplementary Figures 3–5). These results are consistent with the decrease in thickness and density of stress fibers and are often associated with mechanical quiescence in fibroblasts.

To further analyze the mechanical effect of the GPER-mediated decrease in actin stress fibers, we characterized cell (cytoskeletal) stiffness in response to G1 treatment. Cytoskeletal stiffness is dependent on the structure and composition of the actin cytoskeleton and a critical determinant of the cells’ ability to maintain tensional homeostasis, migrate, and deform (Bruckner and Janshoff, 2015; Lautscham et al., 2015). To determine the Young’s modulus of HFFs, we used AFM employing a cantilever with a 15-μm diameter polystyrene bead attached to probe cells in regions distant from the nucleus (Figure 1G). We observed that control HFFs showed a Young’s modulus of 5.6 ± 0.5 kPa (mean ± SEM, *n* = 55 cells), a value within the expected range for fibroblasts (Solon et al., 2007). The Young’s modulus was significantly reduced to 1.1 ± 0.2 kPa (mean ± SEM, *n* = 41 cells) in HFFs treated with G1. When the GPER antagonist G15 was used in conjunction with G1, the Young’s modulus was significantly greater at 5.7 ± 0.3 kPa (mean ± SEM, *n* = 78), not significantly different from control HFFs (Figure 1H), indicating that GPER activation is essential in modifying the rheological properties of the cell. Knockdown of GPER *via* siRNA or expression of constitutively active RhoA similarly exhibited cytoskeletal stiffness at levels comparable to control. These results indicate that GPER modulates not only the composition of the actin cytoskeleton but also its mechanical properties.

### GPER Activation Modulates Focal Adhesion Assembly and Turnover

**FIGURE 2:**
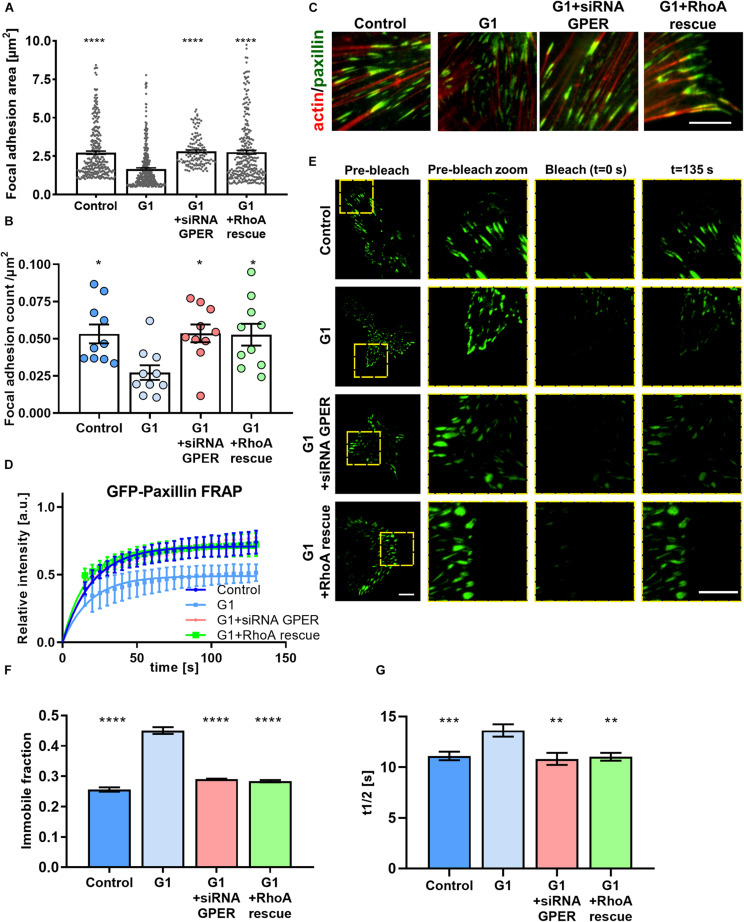
GPER activation regulates the size and dynamics of focal adhesions in HFFs. **(A)** Quantification of paxillin-based focal adhesion area and **(B)** number (normalized by cell area in μm^2^) for control, G1, G1 + siRNA GPER, and G1 + RhoA rescue with constitutively active RhoA (S188A/Q63L); *n* = 276, 130, 240, and 175 focal adhesions from 15, 16, 21, and 18 cells, respectively. Three experimental replicates. Markers denote significant difference from G1 condition by **(A)** Kruskal–Wallis test and **(B)** ANOVA with Dunnett’s *post hoc* test. **(C)** Representative regions of interest for paxillin (immunostaining, green) and F-actin (phalloidin, red) in HFFs cultured on fibronectin-coated glass. Scale bar represents 10 μm. **(D)** FRAP curves for the recovery of GFP-paxillin in focal adhesions of HFFs; curves represent nonlinear fit, one-phase association; points and error bars represent mean ± SD. **(E)** Representative total internal reflection (TIRF)-fluorescence recovery after photobleaching (FRAP) images of GFP-paxillin focal adhesions in HFFs. Scale bar represents 10 μm. **(F)** Immobile fraction and **(G)** half time of recovery data obtained from fit of FRAP curves in panel **(D)**. For control, G1, G1 + siRNA GPER, and G1 + RhoA rescue, *n* = 97, 102, 47, and 47 cells, respectively. Histogram bars represent mean ± SEM, where present, dots represent individual data points. Three experimental replicates. Markers denote significant difference from G1 condition by ANOVA with Dunnett’s *post hoc* test, *0.01 < *P* < 0.05, **0.001 < *P* < 0.01, ***0.0001 < *P* < 0.001, ****P < 0.0001.

The actomyosin cytoskeleton links to the extracellular environment through focal adhesions. These membrane-bound protein complexes are signaling hubs that allow the bidirectional communication of cells with the ECM and drive traction force generation and mechanosensing through regulation of actin polymerization, stress fiber assembly, and modulation of myosin activity (Parsons et al., 2010). Using GFP-paxillin–transfected HFFs and total internal reflection (TIRF) microscopy, we observed that focal adhesions were significantly smaller in G1-treated HFFs compared with those in control cells (Figures 2A,C). Similarly, the density of focal adhesions (number of focal adhesions normalized by the cell area) was significantly decreased in cells treated with G1 (Figure 2B), whereas siRNA knockdown of GPER or RhoA rescue abrogated the effect of G1 on both focal adhesion size and density.

Focal adhesions are highly dynamic structures, with formation, growth, and disassembly dependent on cytoskeletal properties such as mechanical tension and cell contractility (Geiger et al., 2009). The application of force to focal adhesions by the cytoskeleton promotes turnover of focal adhesion components such as paxillin (Wolfenson et al., 2010). We used the GFP-paxillin–transfected HFF cells to image focal adhesions combining TIRF with FRAP. A high-power laser is used to photobleach the GFP-paxillin fluorescence signal in a ROI. As focal adhesions turn over, new GFP-paxillin is incorporated into the bleached adhesions. We observed that following photobleaching, G1-treated HFFs showed a reduced recovery rate compared with control cells, with a significant increase in the time to half recovery (the time required to recover half the final fluorescence intensity), as well as an increase in the immobile fraction (Figures 2D–G). In addition, knocking down GPER *via* siRNA or expression of constitutively active RhoA before G1 treatment recovered the focal adhesion dynamics seen in control HFFs, suggesting that the modulation of focal adhesion dynamics is GPER and RhoA dependent. This indicates that focal adhesion turnover is significantly reduced following G1 treatment, hampering the ability for the cell to interact mechanically with its microenvironment.

### GPER Activation Regulates Actin Polymerization and Expression

**FIGURE 3:**
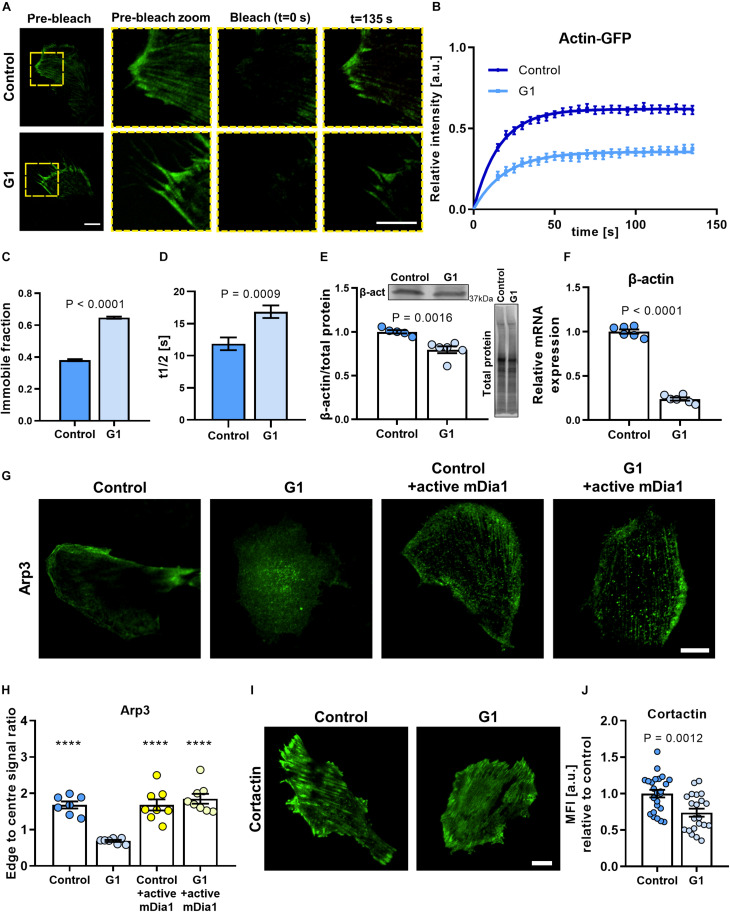
Actin polymerization rate and cell polarization are dependent on the GPER/RhoA axis. **(A)** Representative TIRF-FRAP images of actin-GFP stress fibers in HFFs cultured on fibronectin-coated glass. Scale bar represents 10 μm. **(B)** FRAP curves for the recovery of actin-GFP in HFFs; curves represent nonlinear fit, one-phase association; points and error bars represent mean ± SEM. **(C)** Immobile fraction and **(D)** half time of recovery obtained from fit of FRAP curves in panel **(B)**. For control and G1, *n* = 43 and 30 cells, respectively. **(E)** Western blot quantification of β-actin expression in HFFs. **(F)** Quantification of mRNA levels of β-actin in HFFs. Values are relative to control and normalized to RPLP0 (60S acidic ribosomal protein). Three experimental replicates. **(G)** Representative images of HFFs immunostained for Arp3 in control or G1 conditions with or without constitutively active mDia1 expression. Scale bar is 20 μm. **(H)** Quantification of edge to center Arp3 fluorescence signal ratio. Markers denote significant difference from G1 condition by ANOVA with Dunnett’s *post hoc* test, ****P < 0.0001. For control, G1, control + active mDia1, and G1 + active mDia1, *n* = 31, 30, 29, and 34 cells across seven, seven, eight, and eight experimental replicates, respectively. **(I)** Representative images of HFFs transfected with cortactin-GFP. Scale bar is 20 μm. **(J)** Quantification of cortactin-GFP. MFI, mean fluorescence intensity (expressed in arbitrary units). Scale bar = 20 μm. Histogram bars represent mean ± SEM; dots represent individual data points. *n* = 25, three experimental replicates. *t* test *P* values provided on the graphs.

The ability of cells to rapidly assemble and remodel actin filaments is critical for a variety of dynamic processes, including migration and contraction, as well as the ability to sense and respond to mechanical stimuli (mechanosensing). To assess the effect of GPER activation on actin kinetics, we used actin-GFP to visualize actin filaments in living HFFs with FRAP to quantify actin polymerization rate (Figure 3A).

We measured the fluorescence intensity over time and observed that the fluorescence recovery rate was significantly reduced for cells treated with G1 compared with control HFFs (Figures 3A,B). The time to half recovery was significantly increased from 12 ± 1 (mean ± SEM, *n* = 43) in control HFFs to 17 ± 1 s (mean ± SEM, *n* = 30) in G1-treated cells (Figure 3D), indicating slower recovery and impaired actin polymerization rate with GPER activation. In addition, analysis of the immobile fraction (i.e., the fraction of the fluorescence intensity that is not recovered after bleaching) revealed similar results (Figure 3C), with G1-treated HFFs presenting a significantly higher immobile fraction (65 ± 0.7%, mean ± SEM, *n* = 43 cells) compared with control cells (38 ± 0.6%, mean ± SEM, *n* = 30 cells). These results suggest that GPER activation impairs actin mobility and polymerization kinetics, limiting the ability for the cell to remodel its actin cytoskeleton.

To investigate if the GPER-mediated decrease in actin polymerization rate affected the overall synthesis of β-actin monomers in cells, we quantified the expression of β-actin at the protein and gene levels. β-Actin is the main monomeric form of cytosolic actin, and its expression is critical to the integrity of the cytoskeleton. Interestingly, the expression of β-actin protein was significantly downregulated in G1-treated HFF cells compared with control (Figure 3E and Supplementary Figures 6A,B), a result that was recapitulated in MEFs (Supplementary Figures 6C–E). We also observed a pronounced decrease in the levels of mRNA for β-actin in G1-treated HFFs compared with control (Figure 3F). Conversely, values comparable to controls were observed when G1 treatment was carried out in the presence of the selective GPER antagonist G15 or with siRNA knockdown of GPER (Supplementary Figure 7). Taken together, these results suggest that GPER activation downregulates actin expression either directly or through a negative regulatory feedback in which a reduced actin polymerization rate transcriptionally suppresses the synthesis of β-actin monomers.

### GPER Modulates Cell Polarization in a mDia-Dependent Manner

Another hallmark of mechanically active fibroblasts is the development of a polarized morphology characterized by an increased aspect ratio and an asymmetric distribution of the actin cytoskeleton. Polarization is accompanied by the formation of ventral stress fibers at the trailing edge and actin-rich locomotion structures such as filopodia, lamellipodia, and invadopodia at the leading edge. These structures enable the cell to spread and to probe the mechanical properties of its microenvironment and are thus critical for directed cell migration (i.e., haptotaxis, durotaxis) and mechanosensing (Wu et al., 2012; King et al., 2016; Oakes et al., 2018).

Arp3 is an actin-binding protein that nucleates the formation of actin branches, a process required for the formation of lamellipodia (Mullins et al., 1998; Buracco et al., 2019). In polarized, mechanically active cells, Arp3 is recruited to stress fibers and localizes around the cell periphery, whereas in mechanically quiescent cells, Arp3 remains dispersed through the cytoplasm. We used immunofluorescence microscopy to assess the distribution of Arp3 and confirmed that, in control HFFs, Arp3 is primarily localized in the cell edge, with preferential distribution in one side of the cell, consistent with the asymmetric extension of lamellipodia in mechanically active cells. Conversely, in G1-treated HFFs, Arp3 localizes more uniformly across the cell body (Figure 3G). Quantification of Arp3 distribution revealed an ∼60% decrease in the ratio between the cell edge and cell center in G1-treated HFFs compared with control (Figure 3H).

Interestingly, when cells expressing a constitutively active form of mDia were treated with G1, we observed no significant change in the distribution of Arp3, which localized preferentially to the cell periphery similar to control cells (Figures 3G,H). These results indicate that the mechanism of GPER-mediated modulation of cell polarization is mDia dependent. A protein of the formin family and a RhoA effector, mDia catalyzes the nucleation of linear actin filaments and promotes actin polymerization. mDia and the Arp2/3 complex have been found to cooperate sequentially to generate lamellipodia by regulating the polymerization of mother actin filaments and the branching of daughter filaments, respectively (Isogai et al., 2015).

To further confirm the regulatory effect of G1 on cell polarization, we analyzed the expression of the actin-binding protein cortactin. When activated, cortactin recruits the Arp2/3 complex to mature actin filaments to promote actin branching (Kirkbride et al., 2011). Consistent with our previous results, we observed a significant (∼26%) decrease in the fluorescence intensity levels of cortactin in G1-treated HFFs compared with the control cells (Figures 3I,J). Taken together, these results suggest that inhibition of the RhoA/mDia axis is central to the GPER-mediated modulation of the actin cytoskeleton.

## Discussion

**FIGURE 4:**
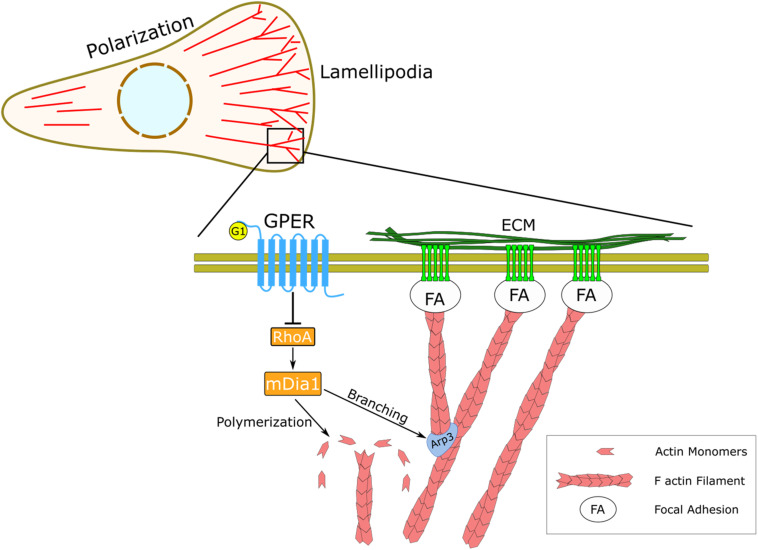
Schematic representation of the regulatory effect of GPER on actin dynamics and mechanosensing. In HFFs, GPER signaling regulates the RhoA/mDia axis, which governs the dynamics of the actin cytoskeleton, including actin filament polymerization and actin branching through the Arp2/3 complex. At the cellular level, GPER modulates cell polarization, lamellipodia protrusion, and mechanical interaction between the cells and the ECM through focal adhesions (FA).

The wealth of physiological and pathological roles of rapid estrogenic signaling through GPER underlies the importance of understanding its regulation and downstream signaling effects. As a member of the versatile GPCR family, GPER influences a large range of biochemical signaling pathways. A growing body of evidence highlights the emerging role of GPER-mediated mechanical pathways in health and disease (Carnesecchi et al., 2015; Wei et al., 2016; Cortes et al., 2019b, d). In this work, we present a previously unidentified biomechanical mechanism in fibroblasts by which the ubiquitous transmembrane receptor GPER controls the structure and dynamics of focal adhesion complexes and the actin stress fibers. We found that activating GPER regulates actin polymerization rate and branching through the RhoA/mDia axis and in turn modulates cell polarization in fibroblasts (Figure 4).

Previous studies reported that GPER can regulate cell morphology and focal adhesion size in dermal fibroblasts (Carnesecchi et al., 2015). Here, we recapitulate those results and demonstrate that GPER signaling further regulates the organization and dynamics of the actin cytoskeleton through RhoA and its downstream effector mDia. The ability to polarize in response to mechanical stimuli is fundamental for directed cell migration such as durotaxis or haptotaxis (King et al., 2016; Lachowski et al., 2017) and depends on differential, asymmetric activation of Rho GTPases such as RhoA and Rac1, which in turn orchestrate actin dynamics at the leading edge (Machacek et al., 2009; Cortes et al., 2019a). Accordingly, we found that actin polymerization and the RhoA/mDia system, which are regulated by GPER, are required for cell polarization and mechanosensing, in agreement with previous work that demonstrates that stiffness and haptotactic sensing by lamellipodia relies on RhoA-mediated actin protrusion, branching, and focal adhesion turnover (Puleo et al., 2019) independently from the ROCK/myosin-2 axis (King et al., 2016; Oakes et al., 2018; Matellan and Del Río Hernández, 2019).

The regulation of actin cytoskeletal dynamics by Rho GTPases also plays a central role in collective cell migration, a process that is fundamental in morphogenesis, wound healing, and cancer (Friedl and Gilmour, 2009). Collective cell migration requires coordinated, dynamic reorganization of the actin cytoskeleton and is characterized by the emergence of “leader cells.” These leader cells present distinct lamellipodial protrusions with increased RhoA and Rac1 activity, both of which are indispensable to maintain the leading cell phenotype and to enable collective migration (Reffay et al., 2014; Yamaguchi et al., 2015). Although not analyzed here, Rac1 is another Rho GTPase, which plays a critical role in single and collective cell migration by modulating the formation of lamellipodia through WAVE/Arp2/3 (Ridley, 2015). While a direct effect of GPER signaling on Rac1 has not been demonstrated, Rac1 has been shown to be downregulated by GPER agonists such as 17β-estradiol and resveratrol (Laufs et al., 2003; Azios et al., 2007), suggesting that GPER signaling could act synergistically through RhoA and Rac1 to regulate actin protrusion in single and collective cell migration.

Analysis of actin polymerization rate and expression revealed that both are concomitantly reduced by GPER/RhoA signaling in fibroblasts, pointing toward an unidentified negative feedback pathway. Regulation of gene expression by RhoA-mediated actin polymerization has been previously described in proximal tubular epithelial cells through the myocardin-related transcription factor A/serum response factor (MRTF-A/SRF) axis (Giehl et al., 2015). MRTF-A is normally inactive in the cytoplasm through binding to G-actin monomers. However, when G-actin is recruited to filaments, MRTF-A translocates to the nucleus along with its binding partner SRF (Miralles et al., 2003), a transcription factor that controls the expression of a variety of cytoskeletal genes, including β-actin, talin-1, vinculin, filamin A, and integrin β1, as well as connective tissue growth factor (CTGF) and matrix metalloprotease 9 (MMP 9; Olson and Nordheim, 2010). We hypothesize that the GPER/RhoA-mediated decrease in actin polymerization leads to accumulation of G-actin monomers and inactivation of the MRTF-A/SRF system. Further experiments will be required to elucidate this mechanism and to investigate the ramifications of GPER signaling on SRF-dependent genes.

The broader implications of GPER-mediated mechanotransd- uction events in fibroblasts will need to be established. For example, GPER could affect actomyosin-dependent ECM remodeling directly impacting on the regulation of connective tissue homeostasis in health and disease (Scott et al., 2015). A stiff fibrotic ECM, generated by fibroblasts and fibroblast-like cells, is also a major clinical hallmark of solid tumors, often associated with aberrant mechanotransduction (Paszek et al., 2005; Jaalouk and Lammerding, 2009; Calvo et al., 2013; Chronopoulos et al., 2016; Sarper et al., 2016), and this GPER-mediated mechanism may provide a therapeutic target wherein mechanical deactivation of fibroblasts leads to a reduction in tumor-permissive desmoplasia.

The physiology of many cells depends on generation and perpetuation of a defined mechanical phenotype, which is often altered in disease and therefore targeted by therapeutics. G protein-coupled estrogen receptor, which we reveal to be a new mechanoregulator, has been investigated for its therapeutic effects in diseases such as cancer, cardiovascular disease, and atherosclerosis (Barton and Prossnitz, 2015; Feldman and Limbird, 2017), all of which have been associated with mechanical deregulation in the disease state (Paszek et al., 2005; Jaalouk and Lammerding, 2009). This suggests that therapeutics targeting GPER may also deregulate mechanopathologies in addition to influencing biomechanical signaling.

Our work positions GPER as a key player in regulating cellular mechanotransduction events in fibroblasts. Given that GPER controls the activation of RhoA, which is a molecular switch highly conserved across species that controls the dynamics of the actin cytoskeleton, and numerous transduction pathways in eukaryotic cells, our findings lay the ground for further investigation on how GPER-mediated changes in the cytoskeleton may control other processes in cells such as adhesion, spreading, migration, membrane protrusion, endocytosis, phagocytosis, and organization of the actin rings at the end of mitosis among many others.

## Data Availability Statement

The raw data supporting the conclusions of this article will be made available by the authors, without undue reservation.

## Author Contributions

DL and ADRH designed the project. DL, EC, and AR performed the experiments and analyzed the data. SDT performed Western blot experiments under the supervision of DAL. DL, CM, and ADRH wrote the manuscript with contributions from all authors.

## Conflict of Interest

The authors declare that the research was conducted in the absence of any commercial or financial relationships that could be construed as a potential conflict of interest.
