# Supplementary material for: G Protein-Coupled Estrogen Receptor Regulates Actin Cytoskeleton Dynamics to Impair Cell Polarization
Source: Front Cell Dev Biol. 2020 Oct 22;8:592628. doi: 10.3389/fcell.2020.592628 (PMC7649801; doi:10.3389/fcell.2020.592628)
Supplement: Supplementary file 1 [file Presentation_1.pdf]

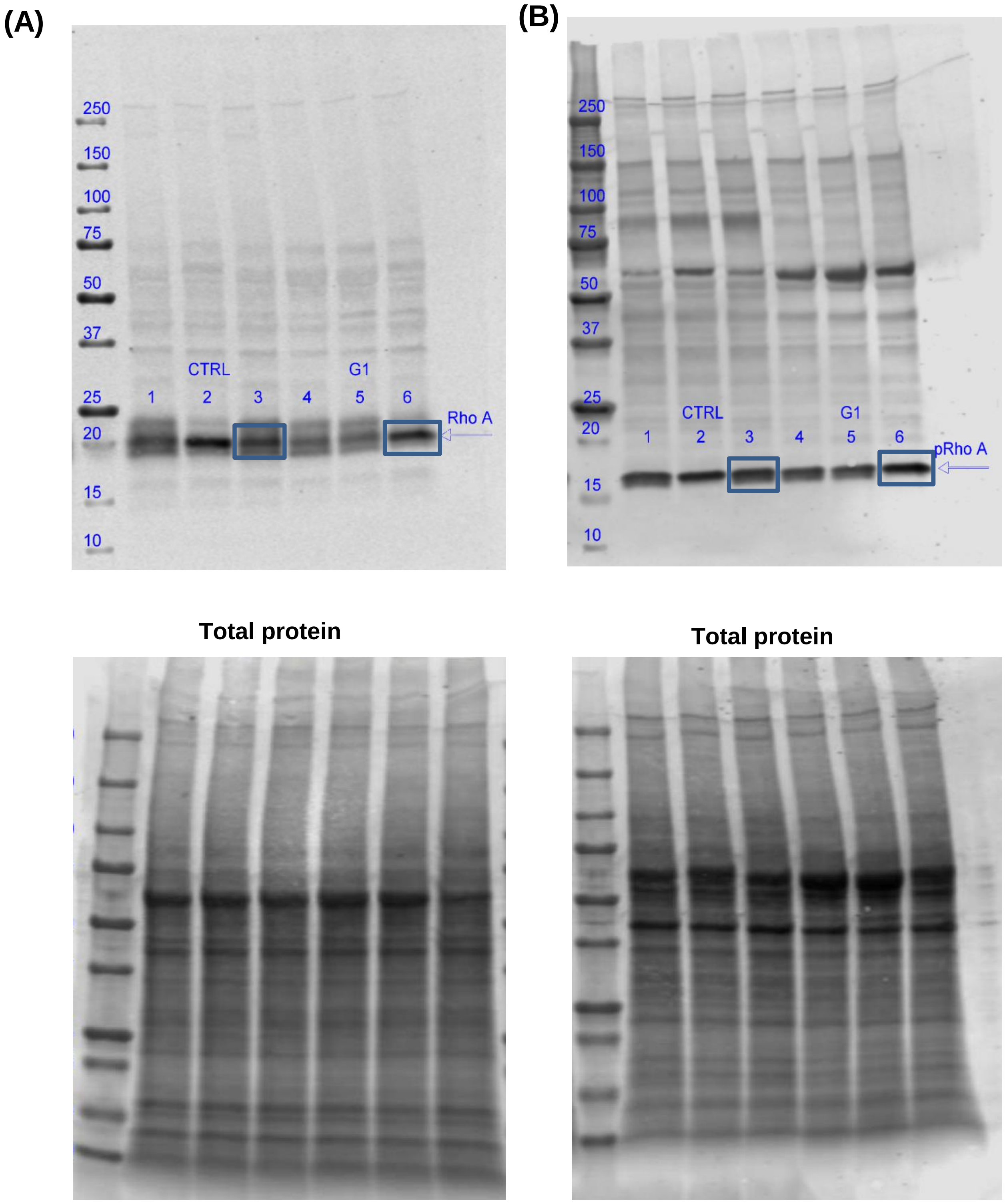

Supplementary figure 1. Images of the full membranes used in Western blots for RhoA (A) and pRhoA (B). HFFs Control and treated with GPER agonist (G1). 9 µg of protein was loaded per lane. Bands presented in figure are indicated by rectangle.

(A)

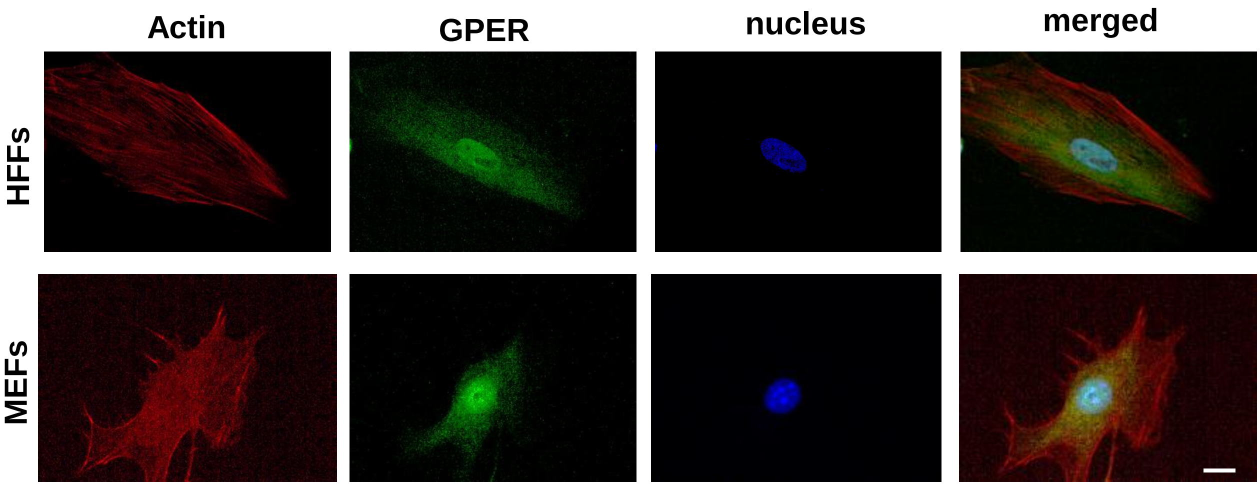

B)

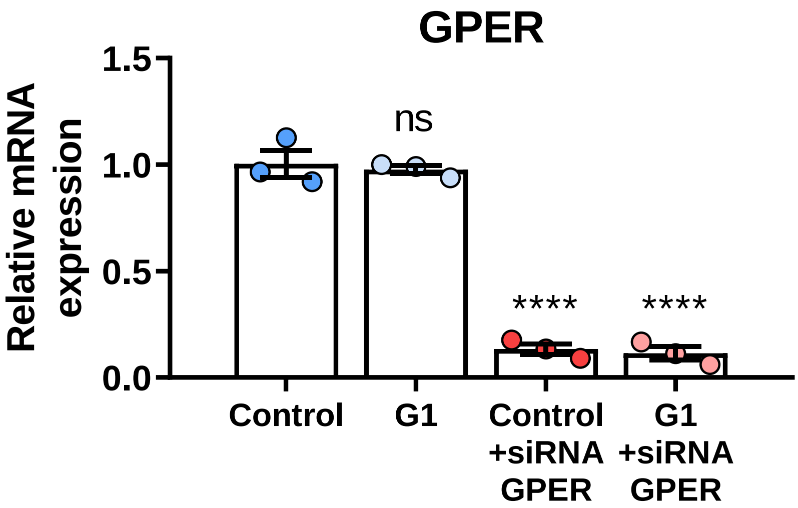

Supplementary figure 2. GPER expression in HFFs and MEFs. (A) Representative immunofluorescent images demonstrating HFFs and MEFs expressing GPER. Scale bar represents 10 µm. **(B)** RT qPCR GPER mRNA expression for HFF control, G1, control + siRNA GPER, G1 + siRNA GPER. Values are relative to control and normalized to RPLP0 (60S acidic ribosomal protein). qPCR data comes from 3 independent experiments. Dots represent individual datapoints, horizontal line marks mean and error bars represent s.e.m. Markers denote significant difference from G1 condition by ANOVA with Dunnett's post hoc test, ns P>0.05, **** P<0.0001.

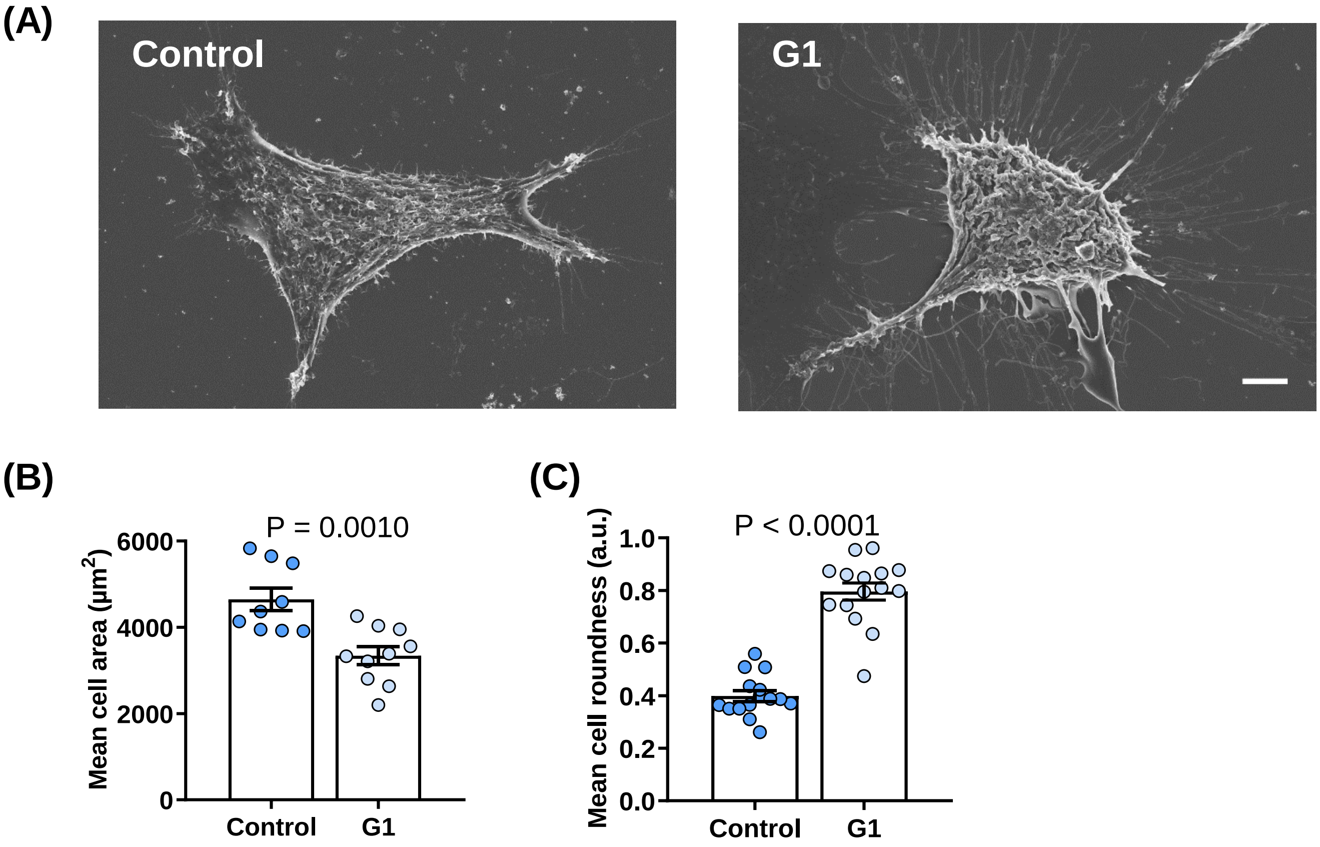

Supplementary figure 3. GPER activation changes the morphology of human foreskin fibroblasts (HFFs). (A) Scanning electron microscopy (SEM) photomicrographs of control and G1 treated HFFs. G1 is GPER agonist. The scale bar is 10 µm. **(B)** Quantification of HFF cell area and **(C)** roundness, n = 10 and 14 cells for control and G1. Dots represent individual datapoints, horizontal line marks mean and error bars represent s.e.m. P – probability value in t-test between control and G1. Three experimental replicates.

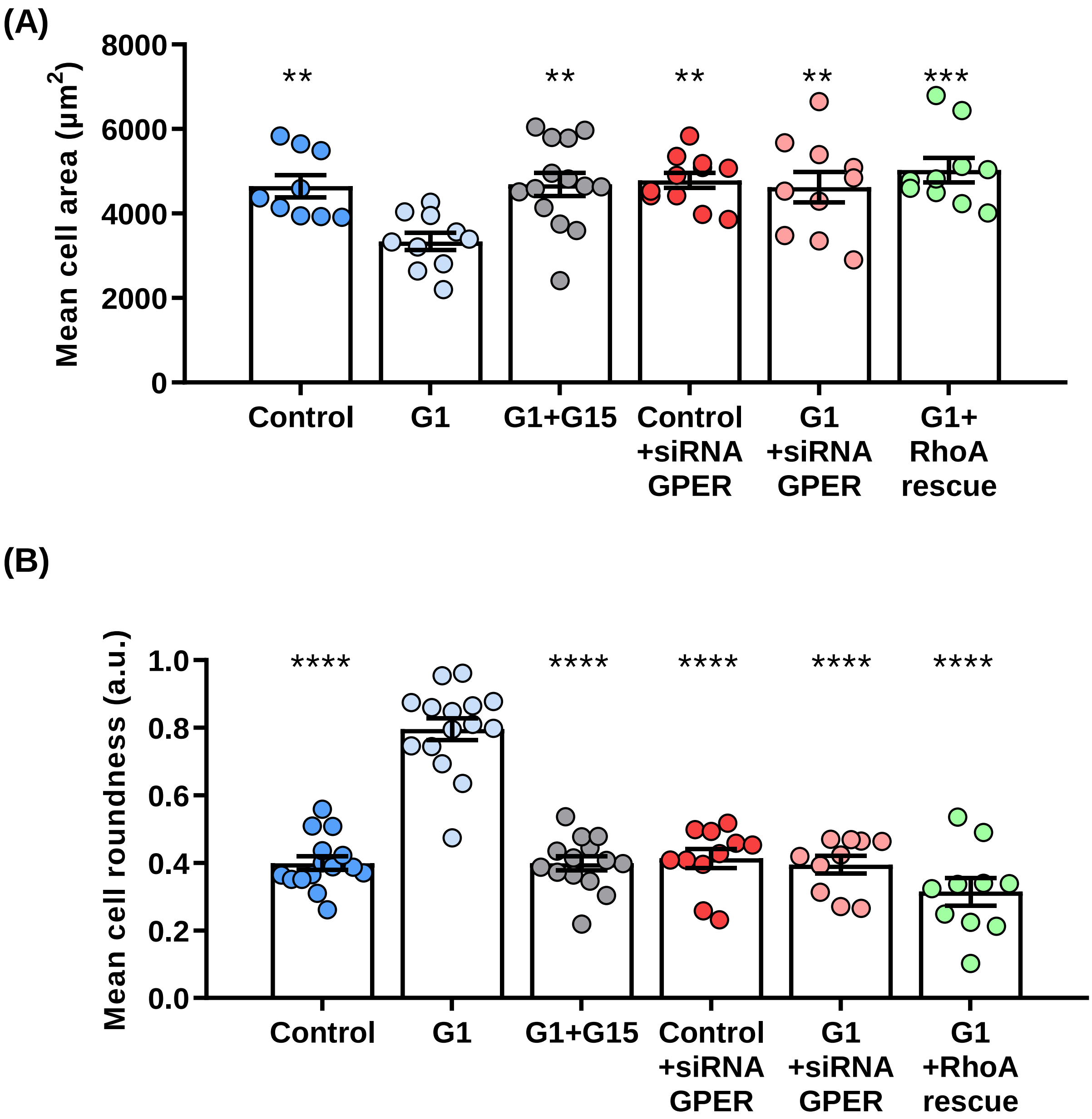

Supplementary figure 4. Characterization of cell morphology in human foreskin fibroblasts (HFFs). HFF cell area **(A)** and roundness **(B)** for control G1, G1+G15, control + siRNA GPER, G1 + siRNA GPER, G1 + RhoA rescue. Histogram bars represent mean ± s.e.m, dots represent individual data points. Three experimental replicates. Markers denote significant difference from G1 condition by ANOVA with Dunnett's post hoc test, ** 0.001<P<0.01, *** 0.0001<P<0.001, **** P<0.0001. Number of cells: 10 control, 10 G1, 10 G1+siRNA GPER, 10 G1+RhoA rescue, 14 G1+G15, and 11 control+siRNA GPER.

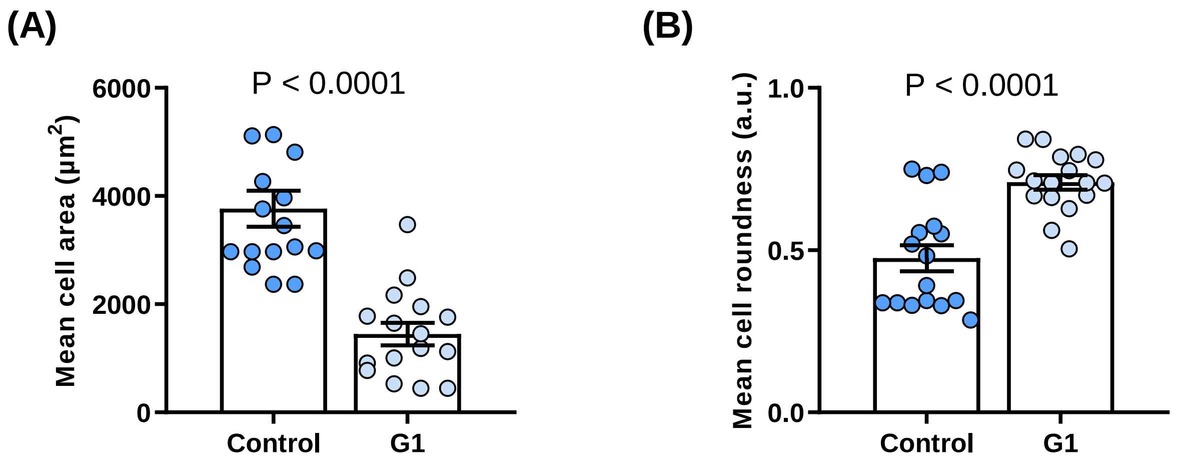

Supplementary figure 5. GPER activation changes morphology in mouse embryonic fibroblasts (MEFs). Quantification of MEF **(A)** cell area and **(B)** cell roundness, n = 16 and 17 cells for control and G1 respectively. Dots represent individual datapoints, horizontal line marks mean and error bars represent s.e.m. P – probability value in t-test between control and G1. Three experimental replicates.

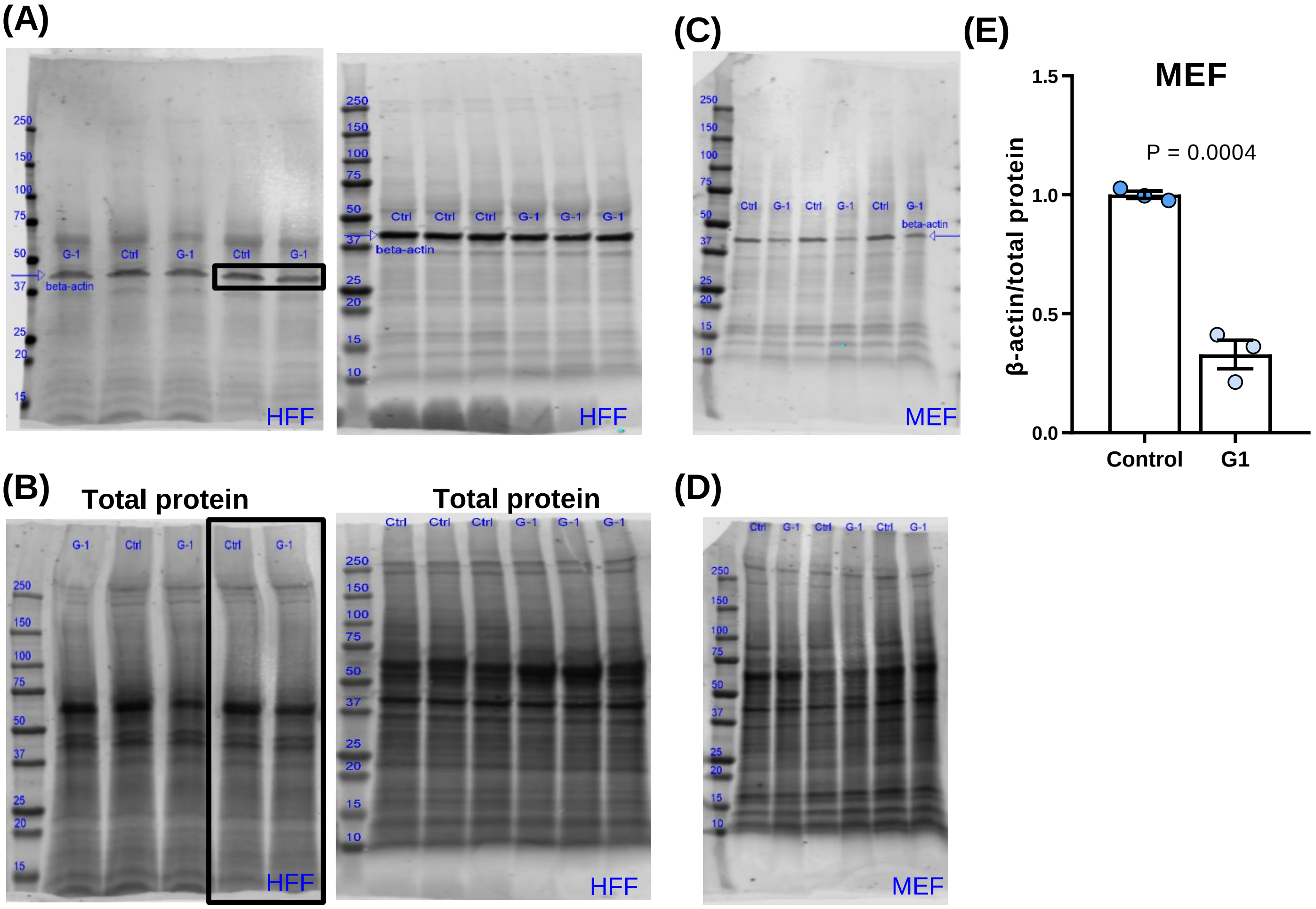

Supplementary figure 6. Full membranes for Western blot of β-actin (A) and total protein (B) in HFFs for blot presented in Fig. 3. Full membranes for Western blot of β-actin (C) and total protein (D) in MEFs used for quantification of β-actin expression (E). Protein from control (Ctrl) and GPER agonist (G1) treated HFFs and MEFs. 20 µg (A & C), 9 µg (B & D) or 10 µg (C, D) of protein was loaded per lane. Bands presented in figure are indicated by rectangle.

(A)

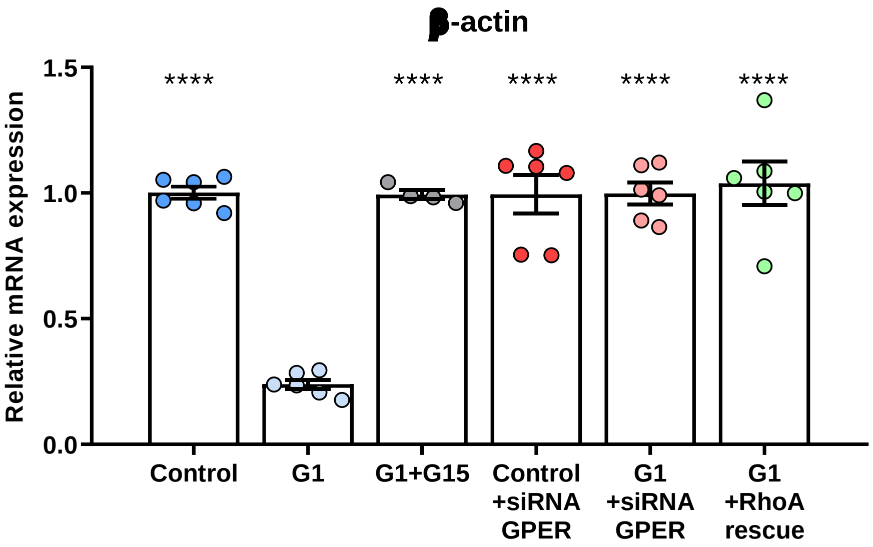

Supplementary figure 7. Actin mRNA expression is dependent on the GPER/RhoA axis. (A) qPCR quantification of mRNA levels of β-actin in HFFs. Values are relative to control and normalized to RPLP0 (60S acidic ribosomal protein). Histogram bars represent mean ± s.e.m, dots represent individual data points. Three experimental replicates. Markers denote significant difference from G1 condition by ANOVA with Dunnett's post hoc test, **** $P<0.0001$
